# Beta-Band Functional Connectivity is Reorganized in Mild Cognitive Impairment after Combined Computerized Physical and Cognitive Training

**DOI:** 10.3389/fnins.2016.00055

**Published:** 2016-02-29

**Authors:** Manousos A. Klados, Charis Styliadis, Christos A. Frantzidis, Evangelos Paraskevopoulos, Panagiotis D. Bamidis

**Affiliations:** ^1^Medical Physics Laboratory, Faculty of Health Sciences, Medical School, Aristotle University of ThessalonikiThessaloniki, Greece; ^2^Research Group for Neuroanatomy and Connectivity, Max Planck Institute for Human Cognitive and Brain SciencesLeipzig, Germany

**Keywords:** aging, brain plasticity, cognitive training, electroencephalography, mild cognitive impairment, graph theory, physical exercise, resting states

## Abstract

Physical and cognitive idleness constitute significant risk factors for the clinical manifestation of age-related neurodegenerative diseases. In contrast, a physically and cognitively active lifestyle may restructure age-declined neuronal networks enhancing neuroplasticity. The present study, investigated the changes of brain's functional network in a group of elderly individuals at risk for dementia that were induced by a combined cognitive and physical intervention scheme. Fifty seniors meeting Petersen's criteria of Mild Cognitive Impairment were equally divided into an experimental (LLM), and an active control (AC) group. Resting state electroencephalogram (EEG) was measured before and after the intervention. Functional networks were estimated by computing the magnitude square coherence between the time series of all available cortical sources as computed by standardized low resolution brain electromagnetic tomography (sLORETA). A statistical model was used to form groups' characteristic weighted graphs. The introduced modulation was assessed by networks' density and nodes' strength. Results focused on the beta band (12–30 Hz) in which the difference of the two networks' density is maximum, indicating that the structure of the LLM cortical network changes significantly due to the intervention, in contrast to the network of AC. The node strength of LLM participants in the beta band presents a higher number of bilateral connections in the occipital, parietal, temporal and prefrontal regions after the intervention. Our results show that the combined training scheme reorganizes the beta-band functional connectivity of MCI patients. ClinicalTrials.gov Identifier: NCT02313935 https://clinicaltrials.gov/ct2/show/NCT02313935.

## Introduction

Age-related neural changes closely associate with cognitive decline in various domains (e.g., long term memory, working memory; Buckner, 2004) and are linked with cerebral structural (Raz et al., 2005), and functional (Geerligs et al., 2015) changes. Consequently, brain function is susceptible to pathological deterioration along the continuum of mild cognitive impairment (MCI), and *Alzheimer's* disease (AD; Fratiglioni et al., 2004; Woodard et al., 2012). The MCI/AD continuum exhibits reduced level of functional communication between distant brain regions and altered patterns of functional brain organization (Bokde et al., 2009).

Burgeoning neuroscientific evidence corroborates that brain abilities are in fact malleable and plastic until the late adulthood (Erickson et al., 2007; Gutchess, 2014). The neuroplasticity changes induced in brain structure and function strongly depend on new (e.g., cognitive, physical, or social) experiences (Maguire et al., 2000; Münte et al., 2002; Draganski et al., 2004; Paraskevopoulos et al., 2012, 2014). Adapting new and positive lifestyles that can potentially aid the elderly cope with the consequences of cognitive decline, may stimulate the brain to create new neural pathways or reorganize existing ones, fundamentally altering how information is processed and compensating for the changes that eventually cause cognitive decline.

Indeed, non-pharmacological interventions in lifestyle have become popular for MCI patients (Petersen et al., 2001; Petersen, 2004). In contrast, pharmacological therapies are non-existent for MCI patients since they do not meet the current criteria for clinically probable AD. Nevertheless, the annual conversion rate of MCI patients to dementia is alarming (~12%), as it is much higher than the one for cognitively healthy seniors (~1–2%; Petersen et al., 1995). A significant percentage (~80%) of the current MCI patients is estimated to convert to dementia within the following five years (Petersen et al., 2009), but then again MCI patients may even reverse back to healthy brain function (Petersen et al., 2001; Larrieu et al., 2002).

A variety of developed interventions aim to promote healthy brain function and plasticity by usually engaging seniors into computerized physical, and/or cognitive training (Colcombe and Kramer, 2003; Busse et al., 2009; Tardif and Simard, 2011; Bamidis et al., 2014). Such interventions allow for the longitudinal evaluation and monitor of the cognitive status of aging populations and may aid in the development of diagnostic biomarkers that can identify early signs along the continuum of MCI and AD-related pathology (Clark et al., 2008; Woodard et al., 2010; Sperling et al., 2011; Styliadis et al., 2015a). Importantly, longitudinal studies may provide insight toward understanding MCI-related brain plasticity associated with unique types of interventions.

The benefits of two factors related to lifestyle, physical exercise and cognitive training, on cognitive performance of aging populations have been amply demonstrated (Bamidis et al., 2014). The growing trend toward cost-effective interventions calls for physical exercise occurring simultaneously or sequentially in the context of cognitive challenges. This scheme can induce more stable neural and cognitive benefits on aging populations in comparison to either physical activity or an enriched environment alone (Oswald et al., 2006; Fabel and Kempermann, 2008; Fabel et al., 2009; Anderson-Hanley et al., 2012; González-Palau et al., 2014).

A previous study from our group explored the effects of combined cognitive and physical training on resting state brain activity of MCI patients in comparison to the effects of solely cognitive or physical training as well as in comparison to active and passive control groups (Styliadis et al., 2015b). The combined intervention induces beneficiary neuroplasticity changes (i.e., decrease delta and theta rhythms in the precuneus/posterior cingulate cortex) across MCI patients and these changes correlate with cognitive status improvement (Styliadis et al., 2015b).

The present study aims at expanding our understanding on the functional benefits of combined training by investigating the role of regional functional connectivity in relation to the MCI condition. Investigating the functional characteristics of the cortex at rest (i.e., neuronal activation patterns and connectivity among regions) in relation to various electroencephalogram (EEG) oscillatory frequency bands is an adequate method which can extract global and local properties characterizing pathological function. Several resting state networks (RSNs) such as the default mode network (DMN), the somatomotor network (SMN) and the dorsal attention network (DAN; Mantini et al., 2007) are considered crucial for cognitive function maintenance. For instance, the DMN's functionality disruption is correlated with amnestic MCI (aMCI; Garcés et al., 2014) and AD (Greicius et al., 2004), and is thus related to the severity and the progression of neurodegeneration (Petrella et al., 2011).

Given that cognitive functions are driven by the interplay of distributed brain areas in large-scale networks (Bressler and Menon, 2010), they cannot be adequately approached by univariate or bivariate models. The brain's complexity formed by the aforementioned interplay represents multivariate relationships best approached via the human connectome. An efficient methodology for studying the human connectome is graph theory which has been widely used to characterize complex brain networks, making it highly suitable to investigate altered connectivity in aging (Nakagawa et al., 2013), neurodegeneration (Zhou et al., 2012) and non-pharmaceutical interventions (Hampstead et al., 2011). Nevertheless, it remains unclear how the human connectome can be altered in response to combined physical and cognitive training among the older adults who experience greater than normal rate of age-related cognitive decline.

In light of the aforementioned research, the current focus is on investigating the functional component of the human connectome at rest that provides insight toward understanding MCI-related brain plasticity, associated with combined cognitive and physical interventions. We hypothesize that the combination of physical and cognitive training will slow down the typical MCI alterations in EEG oscillations, due to their additive beneficial role in enhancing neuroplasticity (Oswald et al., 2006; Fabel and Kempermann, 2008; Fabel et al., 2009; González-Palau et al., 2014).

Herein we present an attempt to identify reliable electrophysiological indices, based on multivariate models, which are able to capture the combined training effects. Fifty MCI patients were divided into two equally distributed groups. The first group trained using a combination of physical and cognitive exercises, while the second one served as an active control group. EEG data were recorded in eyes closed resting condition, before and after each intervention. A new methodology based on group characteristic networks was employed in order to assess the effects of both interventions, in a pre/post manner. The network's density was used as an index for assessing network organization, while the nodes' strength are analyzed in order to reveal which cortical areas are responsible for these effects.

## Materials and methods

### Participants

This study involves 50 (12 male) right handed MCI individuals (mean age = 68.76; SD = 5.89) (Table 1). Each participant went through a detailed neuropsychological examination, which was a prerequisite for the Long Lasting Memories (LLM) project (http://www.longlastingmemories.eu/). This examination took place 1–14 days prior to the intervention onset. The participants were divided into two equally populated groups (25 participants per group) and followed distinct training schemes of the LLM project. The protocol was approved by the Bioethics Committee of the Medical School of the Aristotle University of Thessaloniki, as well as, the Board of the Greek Association of *Alzheimer's* Disease and Related Disorders (GAADRD). Prior to neurophysiological acquisition, each participant received detailed information regarding the study and it was made clear to them that they could terminate the experiment at any time without the need to provide any justification for their decision (no one did). Then, they provided written informed consent. The LLM project was conducted in accordance with the Helsinki Declaration for Human Rights.

**Table 1:** **Subject Pool (Means ± SDs) and training type details**.

	**LLM**	**AC**
No. of subjects	25	25
No. of males/ratio	6 (24%)	6 (24%)
Age	69.60 ± 5.20	67.92 ± 6.40
MMSE	26.04 ± 1.15	25.64 ± 1.26
MOCA	23.40 ± 2.40	23.32 ± 1.99
GDS	1.52 ± 1.45	1.92 ± 2.18
yoe	7.80 ± 2.97	7.04 ± 2.79
Intervention details	PT and CT	Watching documentaries on YouTube
Sessions	Up to 10 h/w	Up to 5 h/w
Duration	PT:25.72 ± 6.24 h CT:28.04 ± 6.39 h	25 ± 4.36 h

LLM, combined training; PT, physical training; CT, cognitive training; AC, active control; MMSE, mini mental state examination, (where the range from best to worst performance is 30–0); MOCA, Montreal cognitive assessment (where the range from best to worst performance is 30–0); GDS, geriatric depression scale, (where the range from severely depressed to normal is 15–0); yoe, years of education; h, hour; w, week.

### Neuropsychological examination

The neuropsychological examination involved the evaluation of the participants' generic cognitive status as well as specific cognitive functions (verbal memory, executive functions, independent living, etc.). This is described in detail in a recent study of our group (Bamidis et al., 2015).

### Diagnostic procedure

The diagnosis was performed by a dementia expert neurologist, naïve regarding the treatment each subject received, taking into consideration the neurophysiological as well as the medical examination. MCI patients met Petersen's criteria (Petersen et al., 2001; Petersen, 2004). All MCI participants had a Clinical Dementia Rating score of 0.5 (Hughes et al., 1982).

### Study criteria

Inclusion criteria for the current study were the following: (i) ages ≥ 60 years, (ii) 23 ≤ Mini Mental State Examination (MMSE) score ≤ 27 points, (iii) 19 ≤ Montreal Cognitive Assessment (MOCA) score ≤ 25 points, (iv) fluent language skills, and (v) agreement of a medical doctor and time commitment to the intervention protocol. The exclusion criteria were: (i) unrecovered neurological disorders (i.e., stroke, traumatic brain injury), (ii) severe depression or psychological disorder, (iii) unstable medication within the last 3 months, (iv) severe physical disorder, (v) severe hearing and/or vision deficits not corrected with lens or hearing aids, and (vi) concurrent participation in another study.

### Categorization and matching

The participants of the LLM group followed a training protocol of computerized physical and cognitive exercises. The participants of the Active Control (AC) group underwent a cognitive stimulation protocol consisting of watching a documentary and answering a questionnaire. All training components were matched on parameters such as intensity, sessions, use of computers (see Table 1). The participants of each group were matched on age [*F*_(1, 48)_ = 0.995, *p* = 0.324], years of education (yoe) [*F*_(1, 48)_ = 0.835, *p* = 0.365], male-to-female ratio (both groups' ratio is 6/25), depression as measured by geriatric depression scale (GDS) [*F*_(1, 48)_ = 0.558, *p* = 0.459] as well as cognitive state as screened by the MoCA [*F*_(1, 48)_ = 0.456, *p* = 0.503] and the MMSE [*F*_(1, 48)_ = 1.320, *p* = 0.256] (see Table 1).

### Long lasting memories (LLM) intervention

LLM is an integrated training system that targets both physiological and cognitively-impaired aging populations through the adoption of cognitive (Smith et al., 2009) and/or physical training (Billis et al., 2010; Konstantinidis et al., 2016). It aims at enhancing the independence of senior citizens by improving their quality of life and their functionality. All intervention components were computerized, center-based and under supervision. The combined cognitive and physical training sessions were performed in a pseudo-randomized counterbalanced sequence. The details of each training intervention are described in detail in (Smith et al., 2009; Billis et al., 2010; Bamidis et al., 2015).

#### LLM trial registration

The trial was registered retrospectively (ClinicalTrials.gov Identifier: NCT02313935). This was a result of strict project timeline but also unclear areas of responsibility in the project (as the trial did not involve any medicinal products covered by Directive 2001/20/EC, guidelines from the European Medicines Agency and Eudra CT in specific, indicated that there was no legal obligation from the sponsor to register it into a trial database).

#### Cognitive training (CT)

The CT component of LLM is a Greek localization product of the Brain Fitness software (Posit Science Corporation, San Francisco, CA, USA). It employs auditory stimuli forming six exercises of user-adaptive difficulty level. Each exercise lasted approximately 15 min. Each CT session consisted of four out of six exercises with an overall duration of 1 h. CT was performed for 1 h per day, three to 5 days per week during a period of 8 weeks. CT targeted auditory processing and working memory (Mahncke et al., 2006b). Details on the benefits of auditory training on age-related cognitive decline are discussed elsewhere (Mahncke et al., 2006a).

#### Physical training (PT)

The PT component of LLM, FitForAll (FFA; Billis et al., 2010), is an elderly-tailored environment (Konstantinidis et al., 2016) where physical exercise is blended by games (exergaming). It employs cost effective, portable and easy to use equipment (Nintendo Wii, Wii remote and Wii balance-board) so as to facilitate an enjoyable digital training experience. PT was performed for three to five sessions per week 1 h per day during a period of 8 weeks. PT was performed in the context of computer-based games which were appropriately adjusted to the elder's physical status. The games' scenarios targeted body flexibility, balance and strength as well as physical endurance through aerobic training. Each participant had to accomplish 20 min of aerobic exercises, 8–10 resistance exercises, 10 min of flexibility exercises and a set of balance targeted exercises. The warm-up and cool-down processes constituted the initial and final session's components respectively. The effects of combined aerobic and strength exercise which is thought to be the most effective exercise training for improving cognitive function are discussed elsewhere (Colcombe and Kramer, 2003; Snowden et al., 2011; Tseng et al., 2011).

#### Active control (AC)

AC is widely used to control for potential confound factors such as willingness to adopt an active aging profile, computer skills and social interaction (Smith et al., 2009). In the current study, though the participants in AC group were exposed to similar training parameters (e.g., computer use, intensity and duration), they just viewed documentaries on nature, art and history and completed questionnaires about the documentaries (Miron-Shatz et al., 2013). Therefore, it may be regarded as a cognitive stimulation protocol which does not involve any PT.

### EEG recordings and pre-processing

EEG recordings were performed in a dark and sound attenuated room. Participants were seated in a comfortable chair and were instructed to close their eyes and stay calm for 5 min. EEG signals were recorded with 57 active electrodes placed on the scalp according to the 10/10 international system (Fp1, Fp2, F3, F4, C3, C4, P3, P4, O1, O2, F7, F8, T7, T8, P7, P8, Fz, Cz, Pz, TP8, AFz, FCz, CPz, FC1, FC2, CP1, CP2, FC5, FC6, CP5, CP6, Fpz, Oz, F1, POz, F2, C1, C2, P1, P2, AF3, AF4, FC3, FC4, CP3, CP4, PO3, PO4, F5, F6, C5, C6, P5, P6, FT7, FT8, TP7). A1 and A2 served as the reference, while the montage used was the linked earlobes. Four ocular electrodes were used, two positioned in the outer canthi of each eye and another two above and below the left eye. The first couple was used to form the bipolar signal for horizontal electrooculogram (EOG) while the second one formed the bipolar signal for the vertical EOG. The signals were amplified and digitized at 500 Hz.

During the pre-processing stage, the EEG signals were filtered between 0.5–45 Hz using the EEGLAB's built-in function for basic FIR filter. This uses a two-way least-squares FIR filter in order to correct the phase delay introduced by the filtering (Delorme and Makeig, 2004). In addition, a notch filter was applied in 47–53 Hz to remove the line's noise. The REG-ICA (Klados et al., 2009, 2011) methodology was applied for the removal of ocular artifacts. This methodology was preferred because it removes the EOG artifacts while keeping the EEG signals more intact than other methodologies (Klados et al., 2011). Extended-ICA (Bell and Sejnowski, 1995) was used to decompose filtered EEG signals into ICs. Subsequently, the algorithm proposed by Schlögl et al. (2007) was used to filter only the EOG contaminated ICs, that are detected manually; finally all ICs (cleaned or not) were used to reconstruct the cleaned EEG signals. Afterwards the bad channels were interpolated using the algorithm included in the EEGLAB software (Delorme and Makeig, 2004). As a final measure, three independent observers checked the EEG signals and removed any bad segments (Supplementary Figure 1). Eventually, 20 s of continuous, high quality, artifact-free data were selected for further analysis, while a randomly selected 20 s segments was also used for the validation of the presented results.

### Cortical activity

In this study we employed the methodology proposed by De Vico Fallani et al. (2010) where the Boundary Element Model method (BEM) implemented in the Brainstorm toolbox (Tadel et al., 2011), was used in order to compute our generic head model. BEM computes the four different compartments of the head model (scalp, outer and inner skull, and cortex) on the basis of an average MRI reconstructed by 152 normal MRI scans (MNI template which is part of the FSL toolbox http://fsl.fmrib.ox.ac.uk/fsl/fslwiki/Atlases (Jenkinson et al., 2012)]. BEM computes the aforementioned compartments by a closed triangle mesh with 4 mm triangle side length and limited number of nodes (in our case we used 305 nodes).

EEG records the activity of the cortical dipoles oriented in tangential or radial directions regarding to the scalp surface. Despite that, the variation of the electrical conductivity among the different head compartments leads to the volume conduction problem, which is a very serious drawback for the functional connectivity analysis. To overcome this problem we performed the functional connectivity analysis on the cortical layer. More precisely, we multiplied the imaging kernel extracted by sLORETA [305 × 57] with our EEG signals [57 × 10,000], so as to obtain the cortical signals on the nodes of the cortex, denoted by the triangular mesh extracted by the BEM (Bin et al., 1999; Lithari et al., 2012; Klados et al., 2013).

### Cortical functional connectivity

A graph is a mathematical object consisted of a set of elements (vertices / nodes) that may be linked through binary connections of weighted connections (edges). In our study, the vertices correspond to the position of the estimated cortical dipoles, while the weight of each edge is given by the Magnitude Square Coherence (MSC) value within each pair of vertices. For this purpose, we used the MSC function of MATLAB v. 7.10 (The MathWorks Inc.) with a 50% overlap, based on recent evidence that it is more suitable to model cerebral networks compared to other connectivity metrics (Lithari et al., 2011). The MSC (1) in a particular frequency (*f*) is defined as the square of the cross Power Spectrum Density (PSD) of signals *x* and *y* divided by the product of the PSDs of *x* and *y* respectively.

(1) $$MSC_{xy}\;(f)=\frac{{\left|P_{xy}(f)\right|}^{2}}{P_{xx}(f)P_{yy}(f)}$$

The PSD was estimated using the Welch method (Welch, 1967). The signals were divided into segments containing 50 samples each, and PSD was then computed using the formula:

(2) $$P(f)=\frac{1}{f_{s}L_{s}U}\int_{-f_{s}/2\;}^{f_{s}/2\;}D_{xx}(\rho ){\left|W\left(f-\rho \right)\right|}^{2}d\rho$$

In this formula *D*_*xx*_ is the Discrete Fourier Transform of the signal's correlation sequence *R*_*xx*_, *f*_*s*_ is the digitization rate (here 500 Hz, *L*_*s*_ is the segment length and *U* is a normalization constant ensuring that the PSD is asymptotically unbiased. So from this process we have 50 (25 pre + 25 post) fully connected graphs for the LLM group and for each one of the six frequency bands: delta (0.5–4 Hz), theta (4–8 Hz), alpha1 (8–10 Hz), alpha2 (10–12 Hz), beta (12–30 Hz), gamma (30–45 Hz) and 50 (25 pre + 25 post) fully connected graphs for the AC group and for each of the aforementioned frequency bands.

### Group characteristic graphs

**Figure 1:**
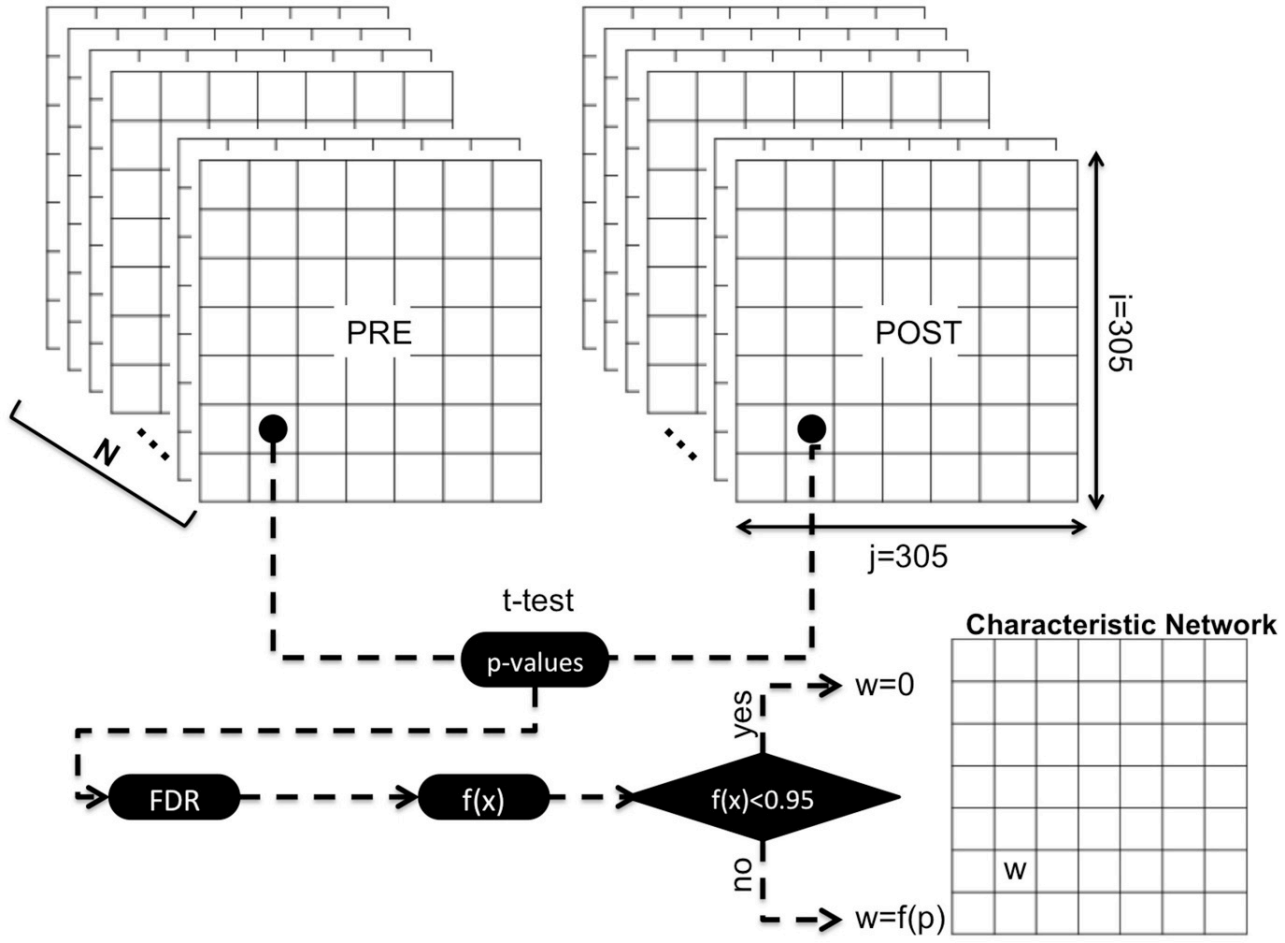
**This figure is a graphical illustration for the extraction of groups' characteristic networks**. For each group and for each frequency band, *N* (*N* = Number of subjects) functional connectivity matrices were obtained for PRE and POST conditions. The values from each (i, j) cell were obtained forming a variable with *N* values for PRE condition and a variable also with N values for POST condition. These two variables where compared using a *t*-test and if their difference is statistically significant (*p*-value < 0.05), after the FDR correction, the (i, j) cell of the characteristic network is equal to the (1–*p*) value, while in the opposite case equals to zero.

The extraction of a unique representative graph for each group was performed according to the “statistical network filtering within populations” approach proposed by De Vico Fallani et al. (2010), which is a novel methodology to extract a connectivity pattern containing exclusively the significant links altered by LLM or AC interventions. According to this methodology we computed the Fisher z-transformation (arc hyperbolic tangent) in order to normalize the MSC value and to approximate the Gaussian distribution. For each frequency band and for each population we formed two 3D adjacency matrices (LLM: 305 × 305 × 25, AC: 305 × 305 × 25), one for the pre and one for the post condition. For each pair of cortical dipoles we compared the distribution of “post” weights with the distribution of “pre” weights using a *t*-test. All the *p*-values were adapted according to the False Discovery Rate (FDR) correction (Benjamini and Hochberg, 1995) and they were reversed (in order the lower *p*-values that denote high significance to correspond to greater graph's weight) using the function: *f*(*x*) = −*x* + 1, where *x* is the *p*-value. Now the interval [0, 0.05] is mapped to the [0.095, 1], while the values that are lower than 0.095 (correspond to non-significant edges) were isolated to 0. (Figure 1).

### Network characteristics density (DEN) and node strength

The density of a graph indicates how many edges are inside the graph divided by the maximum possible number of edges between the vertices of the graph. This definition is used for binary (not weighted) graphs, so we adapted it in order to fit our needs. We define the density of a weighted network (3) as the summary of all weights divided by the maximum possible number of edges between the vertices multiplied by the maximum value of the current connectivity metric (in our case 1) of both groups. So we form the following formula:

(3) $$K=\frac{\sum_{W_{ij}\;\in \;G\text{ and}i\;\neq \;j}W_{ij}}{\left(V*\left(V-1\right)\right)*\text{max}\left(G\right)}\overset{\text{max}(G)\;=\;1}{\Rightarrow }K=\frac{\sum_{W_{ij}\;\in \;G\text{ and}i\;\neq \;j}W_{ij}}{\left(V*\left(V-1\right)\right)}$$

where *V* denotes the number of vertices.

The node's strength is denoted as the sum of the edges' weights attached to the specific node.

## Results

**Figure 2:**
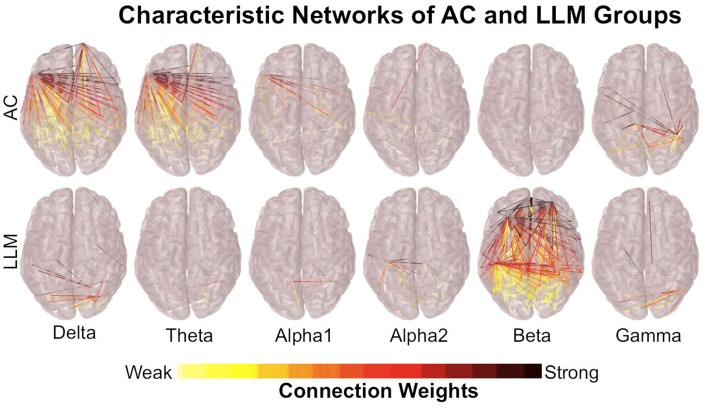
**Cortex plots illustrate the characteristic networks for both groups and for each frequency band**. Each edge represents the inversed *p*-value, extracted by the *t*-test comparison between PRE and POST conditions and corrected using FDR. The LLM group shows stronger effect in the beta-band's network, while AC affects only delta and theta networks. This result is produced by one node in the left fronto-temporal area, which seems to be connected mostly with long distance nodes. Long distance couplings, especially in low frequencies, are probably due to low frequency signaling and not due to synchronous activity, so this effect cannot be interpreted as a solid one.

**Figure 3:**
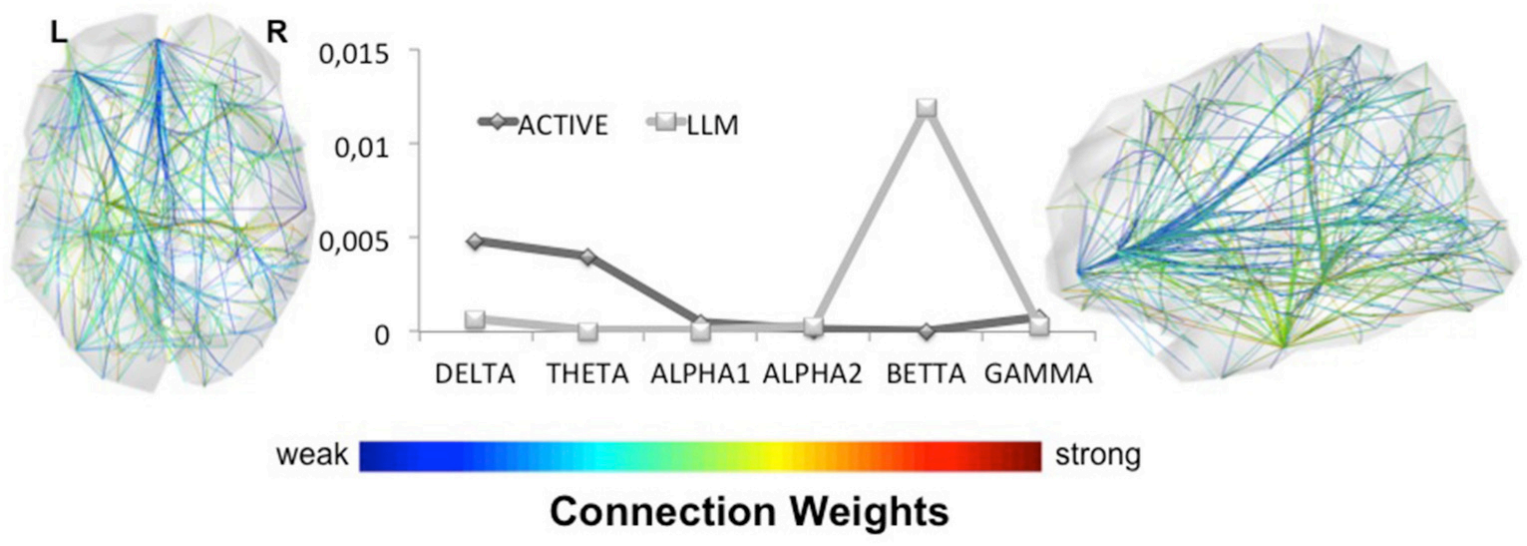
**The line plot illustrates the density of the characteristic networks in all frequency bands**. The highest difference between LLM and AC groups is observed for beta brainwaves. The axial and sagittal views of the LLM's characteristic network reveal its topology. LLM alters beta rhythm, while other bands remain almost intact.

The analysis of the networks obtained by the cortical dipoles was performed in the characteristic networks of each group (LLM, AC) (see Cortical Functional Connectivity). These networks present the statistical contrast between the resting state functional connectivity before and after each intervention. Figure 2 presents the characteristic networks for both groups and for each frequency band, while Figure 3 (Supplementary Figure 2) depicts the line chart of the density among the different frequency bands in the clear (random) EEG segments. The combined (LLM) intervention significantly changes the network's density within the beta activity, while AC training alters only the low spectral components (delta and theta) with a small difference in networks' density. In beta band, LLM's network density is much higher not only compared to the density of the AC group, which is found to be zero, but also compared to the rest characteristic networks. The fact that AC's network density is zero indicates that the pre and post AC networks do not have statistically significant alterations. In the brain plot of Figure 3 and Supplementary Figure 3, the exact LLM's beta network is depicted; the most significantly altered edges are in parietal, temporal and prefrontal regions.

**Figure 4:**
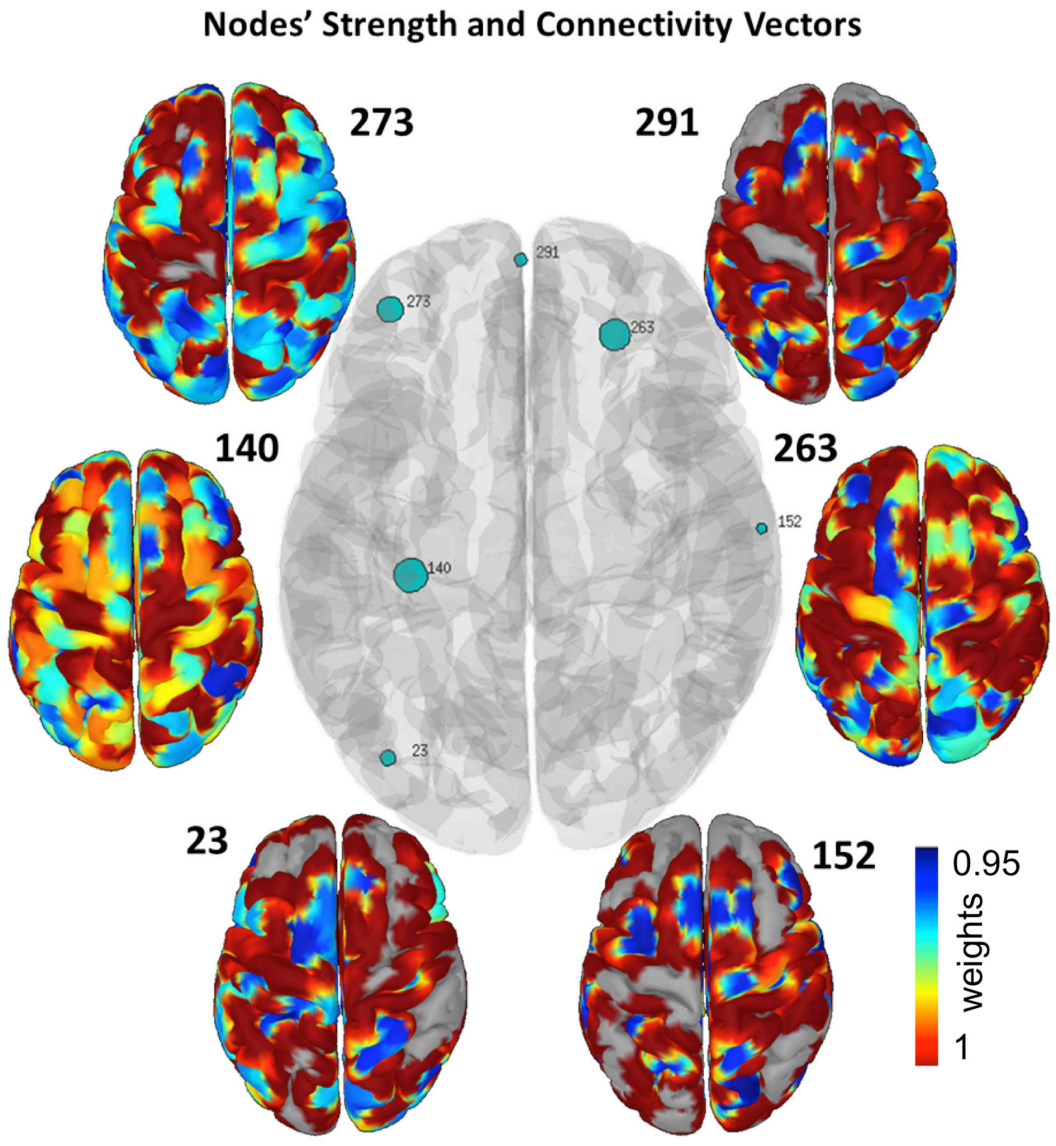
**Central cortex depicts the nodes' strength with green circles, where the size of each circle is in line with each node's strength**. Only nodes with z score higher than 3 are presented. The small cortices indicate the connectivity vector of each node, representing their interconnectivity to the cortex. The robustness of each node's connectivity ranges from minimum (blue areas) to maximum (red areas).

In order to observe the links' functional structure and to conclude which cortical nodes are responsible for the observed differences, we employed the node strength in the beta band network. We chose only to depict nodes that their strength's z-score was greater than 3. The results are depicted in Figure 4, where the size of the marker is in line with the activation of each node, in terms of nodes' strength. Specifically, Figure 4 depicts the connectivity vector of each node while the network's edges represent the *p*-value of the statistically significant difference between the pre and post resting state EEG. The nodes' connectivity is shared with RSNs, i.e., DMN (node 263), DAN (node 291), and SMN (nodes 273 and152). Table 2 lists the node coordinates in both CSC and MNI systems. The most active nodes are in occipital, parietal, temporal and prefrontal regions. Table 3 lists the areas with the most robust connections for the nodes presented in Table 2.

**Table 2:** **Node topography in both CSC and MNI coordinates**.

**Node ID**	**Anatomical area**	**BA**	**CSC Coordinates (Brainstorm) *x, y, z***	**MNI Coordinates (mm) *x, y, z***
23	Middle occipital gyrus	19	−0.061, 0.040, 0.065	−40, −79, 17
140	Medial temporal lobe		−0.009, 0.033, 0.040	−33, −28, −7
152	Postcentral gyrus	2	0.003, −0.065, 0.076	65, −14, 28
263	Middle frontal gyrus	11	0.058, −0.024, 0.033	24, 39, −14
273	Middle frontal gyrus	10	0.065, 0.039, 0.049	−39, 47, 1
291	Medial frontal gyrus	10	0.079, 0.002, 0.034	−2, 61, −13

BA, Brodmann Area; x, left/right; y, anterior/posterior; z, superior/inferior; Significant at p < 0.05, FDR corrected.

**Table 3:** **Areas with the most robust connections for the nodes presented in Table 2**.

	**23**	**140**	**152**	**263**	**273**	**291**
**Most connected areas**	R superior temporal gyrus	R middle frontal gyrus	L occipital fusiform gyrus	L inferior frontal gyrus	L inferior temporal gyrus	R cingulate gyrus
	L superior parietal lobule	R postcentral gyrus	L middle temporal gyrus	L paracentral gyrus	L inferior frontal gyrus	R lingual gyrus
	R middle temporal gyrus	L middle frontal gyrus	L occipital pole	L precentral gyrus	L precentral gyrus	L cingulate gyrus
	R superior parietal lobule	R brain-stem	R superior temporal gyrus	L superior frontal gyrus	L occipital pole	R parahippocampal gyrus

## Discussion

The present study explores resting state EEG functional connectivity characteristics in MCI induced by an eight-week long intervention of combined physical and cognitive training. Changes in connectivity as measured via EEG represent evidence that: (i) the density of the post vs pre network changes significantly within beta rhythm, (ii) the network induced by combined training follows both the dorsal and ventral stream reflecting functional reorganization, and (iii) the nodes' connectivity is shared with RSNs reflecting a functional maintenance of the corresponding neural networks

### The density of the post vs. pre network changes significantly within beta rhythm

Our results suggest that significant changes in density values of the post vs. pre network for the LLM group are mainly found between oscillations in the beta band (14–30 Hz) generated at the level of relatively distant sources. It appears that the neuroplastic processes induced by the combined training in MCI patients, previously reported for cortical sources in another study of our group (Styliadis et al., 2015b), affect also the functional connectivity mainly in the beta band. Our finding for MCI patients replicates the link between beta band power and activities in resting state networks usually reported for healthy adults (Laufs et al., 2003; Mantini et al., 2007). On the other hand, MCI patients compared to healthy counterparts exhibit decreased EEG synchronization and disturbed magnetoencephalography (MEG) functional connectivity within the beta band (Koenig et al., 2005; Gómez et al., 2009). Given that lower beta band synchronization correlates with lower MMSE scores, this frequency band may be of diagnostic importance in dementia, especially in the early stages (Stam et al., 2003). Indeed beta-related biomarkers may be of clinical importance facilitating the AD diagnosis and the neurodegeneration progression (Poil et al., 2013). Having in mind that EEG connectivity and connectomic features can reliably identify early signs along the continuum of MCI and AD-related pathology (Frantzidis et al., 2014b), their information can also be employed to serve as a potential index of gains versus cognitive declines and neurodegenerative burden. Thus, our main interpretation of the post- intervention observed changes in the network indices of the beta band is that they reflect the underlying mechanism of neuroplasticity observed with MCI patients. Finally, the fact that the main effect was found in the beta band may be an indicator that the physical activity drives the improvement in the combined training of the LLM group (Engel and Fries, 2010; Styliadis et al., 2015b).

### The network induced by combined training follows both the dorsal and ventral stream reflecting functional reorganization

The comparison of the characteristic graphs, reveals that the MCI patients who underwent a combined scheme of cognitive and physical training activate a functional network of bilateral activations with prominent contribution of connections between frontal and occipitoparietal brain regions. The contribution of the temporal sources (medial temporal lobe) is smaller but still significant as there are shared connections with frontal sources. As evidenced from our results, the functional network induced by combined training seems to mainly follow a dorsal stream but also the ventral at a lesser degree. The dorsal stream, which has a functional specificity for the processing of spatial information, is reported to be impaired in AD at a greater degree than the functions related to the ventral stream (Mendez et al., 1990). Specifically, a task related functional Magnetic Resonance Imaging (fMRI) study on AD patients showed hypoactivation in the dorsal stream and compensatory recruitment of remote brain areas such as the fusiform gyrus which is located along the ventral stream (Prvulovic et al., 2002). On the other hand, a visuospatial task related fMRI study revealed enhanced activation in the dorsal stream as a treatment effect of therapeutic cholinesterase inhibitors which was found to correlate significantly with improved functioning in terms of activities of daily living (Thiyagesh et al., 2010). In addition, a memory processing MEG study showed that MCI patients when compared to healthy controls exhibit increased bilateral activity in the ventral stream including the medial temporal lobe (MTL; Maestú et al., 2008). Importantly, a cross-sectional and longitudinal MEG study of the same group showed a significant effect in both the ventral and dorsal streams in MCI patients who progressed to dementia and interpreted this as a compensation for the loss of efficiency in memory networks which was absent in AD patients after a period of 2, 5 years (Maestú et al., 2011). Compensatory mechanisms occur early in AD progression, mainly prior to the onset of the clinical phase, and support the aging brain function in maintaining a relatively stable functional responsiveness (Frantzidis et al., 2014b). Our finding that the sample of the present study, being at-risk for dementia (MCI patients), show bilateral activations mainly along the dorsal stream as well as along the ventral stream but in a lesser degree could be interpreted as resulting patterns of functional reorganization. Given that the most prominent symptoms of MCI and AD are visuospatial impairments and memory loss, the functional connectivity pattern employed by MCI patients that were consistently stimulated with visual, auditory, and motor information reflects greater efficiency and functional reorganization and is denser than the corresponding network of their control counterparts and can thus be regarded as a neuroplastic outcome induced by the combined training.

### The nodes' connectivity is shared with RSNs suggesting a functional maintenance of the corresponding neural networks

The nodes of the functional network exhibit connections with widespread brain areas that can be considered to form well-identified RSNs, which are vulnerable to AD neuropathology as highlighted in the following literature. For instance, node 263 shows connectivity with areas of the DMN (Raichle et al., 2001). Specifically, it is connected with the left inferior frontal gyrus, which is active during both conscious resting-state and working memory or reasoning tasks while being significant for inhibition control (Mazoyer et al., 2001). This node is also connected with a number of left-hemisphere structures, i.e., paracentral, precentral, and superior frontal gyrus. A common characteristic of all the brain regions is that these demonstrate reduced activations in both physiological aging (Salat et al., 2004; Damoiseaux et al., 2008) and pathological aging, i.e., aMCI phase (Sorg et al., 2007) and mild dementia (Wang et al., 2007). An fMRI study by Greicius et al. (Greicius et al., 2004) revealed decreased resting-state activity early in the course of AD in the posterior cingulate and hippocampus suggesting also disrupted connectivity between these regions. Also, Rombouts et al. (2005) used fMRI to reveal less deactivation in resting state activity of MCI patients compared to controls, but more than AD, in the anterior and medial frontal cortex. Node 291 has connections with areas of the DAN (Corbetta and Shulman, 2002). This network node is mainly connected with the cingulate gyrus of both hemispheres as well as with the lingual and the parahippocampal gyrus located on the right hemisphere. Previous studies have demonstrated that AD neuropathology is more prominent on the hippocampus, precuneus, posterior cingulate cortex, and parieto-occipital brain regions (Krüger et al., 2012). The AD neuropathology in the cingulate gyrus is demonstrated through reduced gray matter amount and diminished connectivity (Damoiseaux et al., 2012). The lingual gyrus is an important region involved in cognitive processing and participates in the ventral DMN, while it is also vulnerable to AD neuropathology. Also, there is evidence that the right parahippocampal gyrus is affected early in the AD neuropathology (Pantel et al., 2003) as this structure faces significant atrophy during the MCI phase. DMN and DAN are of particular interest as the former is engaged by internally directed cognition and the latter mediates goal-directed stimulus-response selection, and hence are anti-correlated (Fox et al., 2005). The disturbance of this anti-correlation may be associated with the attention deficits of AD patients (Wang et al., 2007). Nodes 152 and 273 have connection with areas of the SMN (Biswal et al., 1995). Areas of the SMN have crucial role in the modulation of episodic memory, action recognition and spatial navigation (Russ et al., 2003). These findings support the functional reorganization proposed here for MCI patients which seems to affect many resting state network that govern cognition, memory and attention.

### Strengths and limitations

An important advantage of the present study is that both intervention groups received a well-distributed social support to promote the psychological well-being of MCI patients, since positive mood states could influence individual cognition and brain function (Subramaniam and Vinogradov, 2013). It is also of crucial importance that the training group was compared with an active control group and not a no-contact (passive) group. This strengthens the study results by posing a more difficult comparison criterion and eliminating methodological problems due to a passive control group. A limitation of the study is the fact that there was no clinical follow-up on the MCI patents. Thus, outcome still remains unknown; some may progress to develop AD or other dementia while others may remain stable or improve to normal. This limits any further insight on whether the training protocol employed here can be implemented with reliable intensity, frequency and duration to achieve long-lasting cognitive gains. Nevertheless, the current findings are in support to the hypothesis that physical exercise and cognitive stimulation can potentially improve interregional connectivity of brain areas sub serving cognitive performance in cognitively pathological populations (Frantzidis et al., 2014a).

The forward model used for the inverse solution in the present study was generated from a boundary element head model based on the standard MNI-template. This strategy is proposed as the most favorable one in the case that no individual MRIs are available (Vatta et al., 2010) but it still generates limitations that are related with localization inaccuracies in comparison with the individualized head models. A recent comparison of the effect of different forward models in EEG inverse solution (Akalin Acar and Makeig, 2013) revealed that the median localization error of MNI based head model was 5 mm. This should be taken in to account when interpreting the exact localization of the present results, but nevertheless, this should not affect the statistical differences found between conditions in the present study and hence, it would not affect the functional outcome of our results.

## Conclusion

An eight week intense intervention of combined physical exercise and cognitive training induces neuroplasticity changes among older adults who experience greater than normal rate of age-related cognitive decline. Here, we provide evidence that even short training combining cognitive and physical components has the potential to alter the interregional functional connectivity. Thus our findings on the marked signs of functional reorganization induced by combined training reflect enhanced neuronal plasticity. Future connectivity studies of similar results may aim at providing post intervention correlations of functional reorganization changes with cognitive function so as to elucidate the exact beneficial nature of these changes in daily life functioning and further explore the compensatory mechanisms recruited by MCI patients.

## Author contributions

MK and CS are equal first authors. MK performed the connectivity analysis, prepared the figures and contributed to the writing of the manuscript. CS collected the demographics of the study's sample, interpreted the findings and had major contribution to the writing of the manuscript. CF performed data acquisition, pre-processing and reviewed the manuscript. PB supervised the whole project including intervention, recordings and analysis sessions. EP supervised the brain source analysis. EP and PB reviewed the manuscript. PB had substantial contribution to the conception of the intervention.

### Conflict of interest statement

The authors declare that this research was conducted in the absence of any commercial or financial relationships that could be construed as a potential conflict of interest.

## Supplementary material

Supplementary Figure 1 **Preprocessing pipeline of the EEG signals used in this study**.

Supplementary Figure 2 **The density of the networks generated by the randomly chosen segments**.

Supplementary Figure 3 **This figure illustrates the Beta networks of the LLM (left) and AC (middle) groups generated by the randomly chosen segments, while the right one illustrates the distribution of the nodes that have z-score greater than 3 in accordance to the Figure 4**.
