# Supplementary figures and images for: Beta-Band Functional Connectivity is Reorganized in Mild Cognitive Impairment after Combined Computerized Physical and Cognitive Training
Source: Front Neurosci. 2016 Feb 29;10:55. doi: 10.3389/fnins.2016.00055 (PMC4770438; doi:10.3389/fnins.2016.00055)

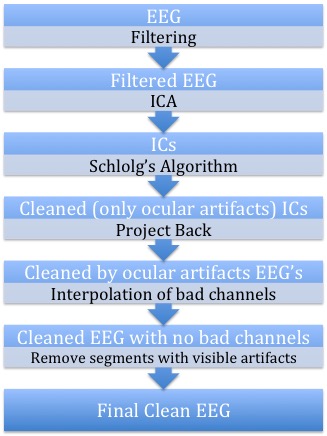

Supplement: Supplementary Figure 1 — Preprocessing pipeline of the EEG signals used in this study. [file Image1.JPEG]

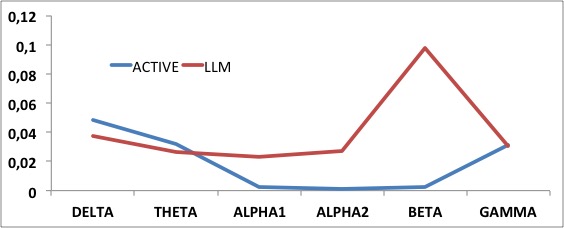

Supplement: Supplementary Figure 2 — The density of the networks generated by the randomly chosen segments. [file Image2.JPEG]

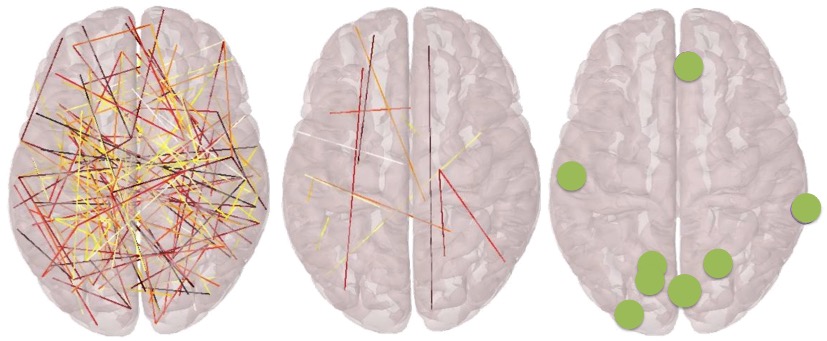

Supplement: Supplementary Figure 3 — This figure illustrates the Beta networks of the LLM (left) and AC (middle) groups generated by the randomly chosen segments, while the right one illustrates the distribution of the nodes that have z-score greater than 3 in accordance to the Figure 4. [file Image3.JPEG]
